# Development of hydrogen sulfide donors for anti-atherosclerosis therapeutics research: Challenges and future priorities

**DOI:** 10.3389/fcvm.2022.909178

**Published:** 2022-08-12

**Authors:** Ye-Wei Yang, Nian-Hua Deng, Kai-Jiang Tian, Lu-Shan Liu, Zuo Wang, Dang-Heng Wei, Hui-Ting Liu, Zhi-Sheng Jiang

**Affiliations:** ^1^Hunan Provincial Key Laboratory for Special Pathogens Prevention and Control, Hunan Province Cooperative Innovation Center for Molecular Target New Drug Study, Institute of Pathogenic Biology, Hengyang Medical School, University of South China, Hengyang, China; ^2^Key Laboratory for Arteriosclerosis of Hunan Province, Hengyang Medical College, Institute of Cardiovascular Disease, University of South China, Hengyang, China

**Keywords:** hydrogen sulfide, donor, atherosclerotic, nanotechnology, drug delivery and targeting

## Abstract

Hydrogen sulfide (H_2_S), a gas transmitter found in eukaryotic organisms, plays an essential role in several physiological processes. H_2_S is one of the three primary biological gas transmission signaling mediators, along with nitric oxide and carbon monoxide. Several animal and *in vitro* experiments have indicated that H_2_S can prevent coronary endothelial mesenchymal transition, reduce the expression of endothelial cell adhesion molecules, and stabilize intravascular plaques, suggesting its potential role in the treatment of atherosclerosis (AS). H_2_S donors are compounds that can release H_2_S under certain circumstances. Development of highly targeted H_2_S donors is a key imperative as these can allow for in-depth evaluation of the anti-atherosclerotic effects of exogenous H_2_S. More importantly, identification of an optimal H_2_S donor is critical for the creation of H_2_S anti-atherosclerotic prodrugs. In this review, we discuss a wide range of H_2_S donors with anti-AS potential along with their respective transport pathways and design-related limitations. We also discuss the utilization of nano-synthetic technologies to manufacture H_2_S donors. This innovative and effective design example sheds new light on the production of highly targeted H_2_S donors.

## Introduction

Hydrogen sulfide (H_2_S) is a colorless gas that smells like rotten eggs and has toxic effects at concentrations approaching 20 ppm. Initial research on H_2_S was largely conducted in the context of elimination of H_2_S waste gas in industrial operations and protection from dangerous gases in wartime (1). H_2_S poisoning is caused by the reaction of H_2_S with trivalent iron in oxidized cytochrome oxidase, which inhibits the function of cellular respiratory enzymes, resulting in cellular hypoxia (2). H_2_S can also inactivate glutathione by coupling with its sulfhydryl group, inducing cell death (3). However, it was only in 1996 that Abe and Kimura (4) published the findings of a seminal investigation on endogenous H_2_S generation and signaling. Consequently, over the next 20 years, the general perception of H_2_S shifted from that of a poisonous gas to a gas transmitter with potential for pharmacological therapy. Carbon monoxide and nitrogen oxide (5) are all similar gas transmitters. All gas transmitters have comparable qualities, such as solubility, free diffusion, and the need for certain enzymes and substrates for production. The characteristics of the three gas transmitters are summarized in Table 1.

**Table 1:** Comparison of common characteristics of the three gas transmitters.

	**Hydrogen sulfide**	**Nitric oxide**	**Carbon monoxide**
Molecular stereogram	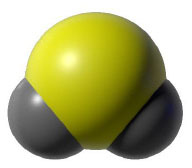	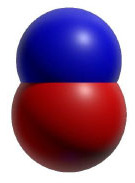	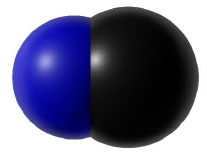
Formula	H_2_S	NO	CO
Solubility (q)	0.289	0.040	0.020
Diffusivity in water	8.6^*^10^−4^cm^2^/min	1.5^*^10^−4^cm^2^/min	1.18^*^10^−4^cm^2^/min
Resources	L/D-cysteine	L-arginine or nitrite	Protohaem IX
Enzymes	CBS, CSE, 3MST/AAT, and DAO	eNOS, iNOS, and nNOS	HO^−1^, HO^−2^, and HO^−3^

* *provide significance times.*

H_2_S has numerous key regulatory effects in AS, including anti-oxidative stress, prevention of endothelial mesenchymal transition, reduction of foam cell production, and modulation of mitochondrial autophagy (6–8). Several studies have demonstrated the cardiovascular benefits of H_2_S in clinical settings. In a randomized controlled, double-blind trial involving 120 hypertensive patients, taurine supplementation significantly decreased the clinic and 24-h ambulatory blood pressure (9). Furthermore, changes in blood pressure were negatively correlated with both the plasma H_2_S and taurine levels in taurine-treated prehypertensive individuals. The potential underlying mechanism is that taurine up-regulates the expression of H_2_S synthase by inhibiting calcium influx in transient receptor potential channels, thereby reducing vascular reactivity. In addition, H_2_S prodrug SG1002 is already being investigated in Phase 1 clinical trials. David J. Polhemus' team conducted a small-sample non-randomized controlled clinical study to show that *in vitro* administration of the H_2_S prodrug, SG1002, can alleviate heart failure. SG1002 reduced the level of brain natriuretic peptide (BNP), and showed no apparent toxic side-effects (10). Thus, H_2_S is a novel cardiovascular disease drug worthy of further research and development.

H_2_S donors are compounds that can release H_2_S when particular trigger conditions are met. Given the features of the gas transmitter, developing an H_2_S donor with high targeting ability and stability during transportation is critical for studying the anti-AS properties of exogenous H_2_S. Moreover, development of suitable H_2_S donor would provide the foundation for future H_2_S anti-AS prodrug research and clinical therapy. In this article, we review the various kinds of donors with anti-AS potential and discuss the pros and cons of each donor design. We also propose the idea of preparing H_2_S donors using nanomolecule technology in combination with chemical synthesis technology. We anticipate that multidisciplinary collaboration will foster the translation of H_2_S anti-AS from basic research to clinical treatment in the future.

## Physical and chemical properties of H_2_S

H_2_S has a molecular weight of 34.08, which is slightly greater than the molecular weight of water. It has a vapor pressure of 2,026.5 kPa/25.5°C, flash point of −50°C, melting point of −85.5°C, boiling point of −60.4°C, relative density of 1.19 (air = 1), and tipping point of 292°C. H_2_S is highly soluble in water; it is also soluble in alcohol, petroleum solvents, and crude oil. It may be hydrolyzed to hydrogen ions, sulfur-hydrogen ions, and sulfur ions in water or plasma using the reaction equations below. The first step: H_2_S+H_2_O=HS^−^ +H_3_O^+^; the second step: HS^−^ + H_2_O=S^2−^+H_3_O^+^. H_2_S is soluble up to 80 mmol/L at typical human body temperature and can be oxidized to sulfides, sulfates, persulfides, and sulfites (11).

## The “multi-dimensional” anti-atherosclerotic effects of H_2_S

**Figure 1:**
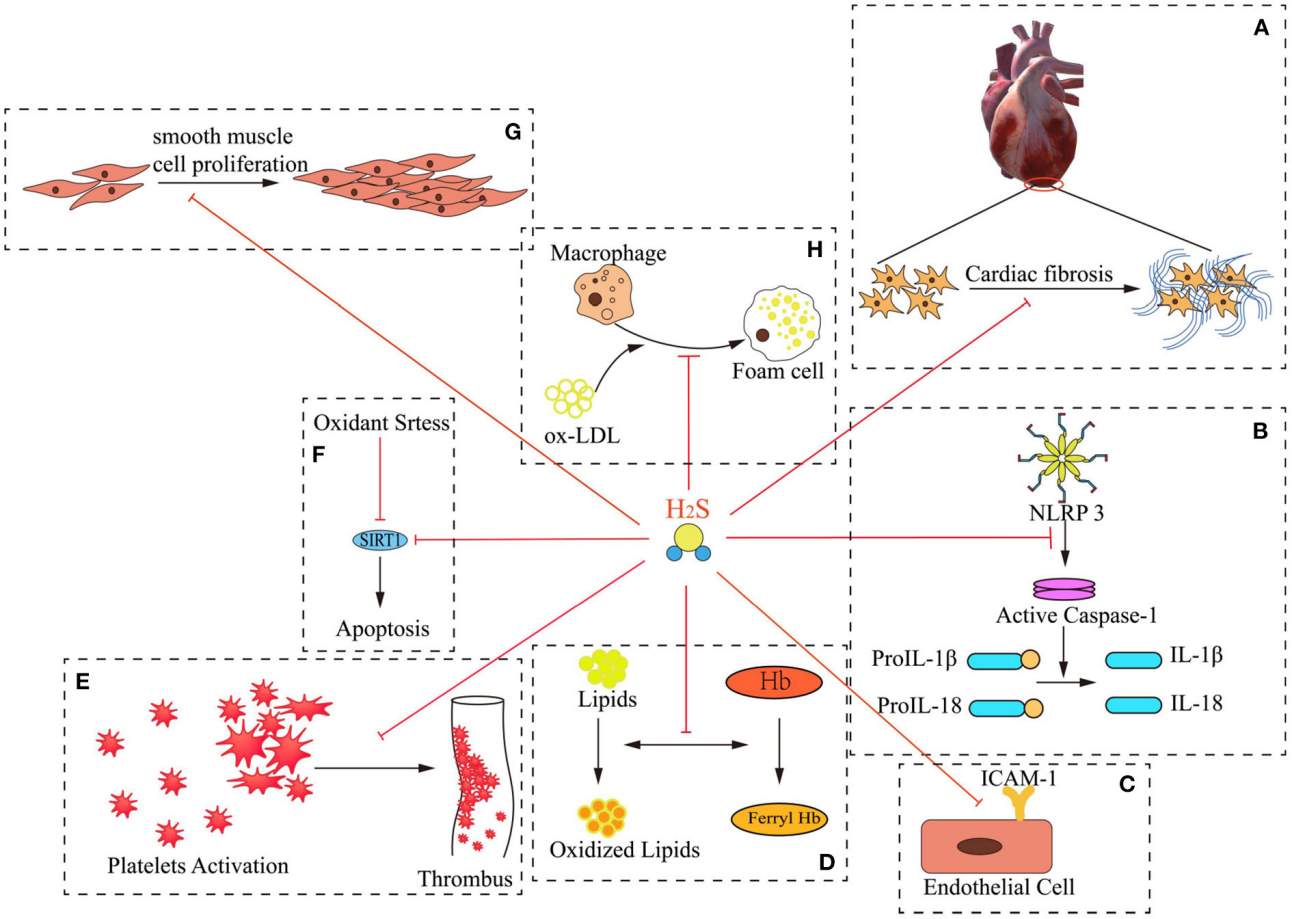
Mechanisms of H_2_S “multi-angle” inhibition of AS. H_2_S exerts numerous critical effects against the pathogenesis of atherogenesis. These include: **(A)** improving myocardial fibrosis; **(B)** inhibiting the production of IL-1β and IL-18; **(C)** inhibiting ICAM-1 expression in TNF-alpha-induced HUVECs *via* the NF-kappa B pathway; **(D)** preventing lipid peroxidation; **(E)** inhibiting intravascular thrombosis; **(F)** protecting against apoptosis under oxidative stress through SIRT1 pathway; **(G)** inhibiting smooth muscle cell proliferation; **(H)** attenuating foam cell formation.

AS is the primary cause of atherosclerotic heart disease, cerebral infarction, and peripheral vascular diseases. Persistent inflammation plays a key role in the pathogenesis of AS (12). The pathogenetic mechanism is complex and involves endothelial dysfunction, leukocyte adhesion and aggregation, lipid plaque deposition, smooth muscle cell proliferation, and extracellular matrix remodeling, among other factors (13). Of note, a large number of studies have demonstrated that H_2_S can protect the cardiovascular system against several elements involved in AS progression, such as by ameliorating myocardial fibrosis; inhibiting IL-1 and IL-18 production; inhibiting ICAM-1 expression in TNF-alpha-induced HUVECs *via* the NF-κB pathway; preventing lipid peroxidation; inhibiting intravascular thrombosis; protecting against apoptosis induced by oxidative stress *via* the SIRT1 pathway; inhibiting smooth muscle cell proliferation; and attenuating foam cell formation (Table 2 and Figure 1). These findings demonstrate the anti-AS potential of H_2_S.

**Table 2:** Anti-AS effect of H_2_S from different mechanisms.

**Hydrogen** **sulfide donor**	**Targets**	**Targeting outcome**	**Biological effect**	**Literature** **sources**
NaSH	SIRT1	Increases SIRT1, activates eNOS and PGC-1α	Anti-oxidant stress	(14)
NaSH	KATP	Inhibits KATP/ERK1/2 pathway, Down-regulates CD36, SR-A and ACAT1 expressions	Inhibits foam cell formation	(15)
NaSH	NLRP3	Suppresses IL-1β and IL-18 release in adipocytes	Inhibits the inflammatory response of adipocytes	(16)
GYY4137	Hb	Inhibits hemoglobin oxidation and prevents lipid peroxidation.	Protects vascular endothelial cells	(17)
GYY4137	Myocardial fibroblasts	Inhibits TGF-β1/Smad2 signal pathway and α-SMA expression	Suppresses EndMT	(18)
S-diclofenac	Smooth muscle cell	Stabilizes p53, p21, p53AIP1 and Bax	Inhibits smooth muscle cell proliferation	(19)
NaSH	HUVECs	Suppresses IκB-α degradation and NF-κB nuclear translocation	Decreases ICAM-1 expression	(20)
ACS-14	Platelet	Activates fibrinogen receptors and increases intracellular cAMP levels	Attenuates arterial thrombus formation	(21)

### H_2_S ameliorates myocardial fibrosis

Diffuse myocardial fibrosis is strongly related with prior cardiovascular events and may result in serious complications. Ambale-Venkatesh et al. (22) used contrast-enhanced cardiac magnetic resonance (CMR) to assess differences in myocardial fibrosis measured at the year-10 examination between participants with and without cardiovascular (CV) events accrued in a large population-based study over a 10-year follow-up period. The findings implied that the prevalence of CV events during the last decade was related with an increased risk of ischemic myocardial scarring on advanced gadolinium-enhanced imaging and greater diffuse interstitial fibrosis as measured by T1 imaging in a multiethnic free-living population.

The effect of H_2_S on fibrosis has previously been examined. Sheng et al. (23) examined human atrial fibroblasts using the BrdU test. The research demonstrated that NaHS at concentrations of 100, 300, and 500 μM inhibited the proliferation of atrial fibroblasts by 33.1 ± 4.2, 43.7 ± 3.1, and 58.4 ± 6.2% respectively. Additionally, they verified the combined inhibitory effects of H_2_S on BKCa and Ito currents in suppressing cellular growth using whole cell patch clamping. Cx43 is intimately linked to myocardial fibrosis, and lower Cx43 expression predisposes to collagen accumulation (24). By partly ligating the rat abdominal aorta, Huang et al. generated a rat hypertrophic cardiomyopathy model. They verified in this experiment that sodium hydride-treated rats had smaller LVMI, cardiomyocyte size and area, and CVF than control rats (25). Additionally, H_2_S has been shown to greatly boost CX43 expression in rat cardiomyocytes. These findings suggest that H_2_S may act as an anti-fibrosis agent *via* increasing CX43 expression.

### H_2_S inhibits the inflammatory response of adipocytes

Adipose tissue is regarded as an important endocrine organ and is known to be involved in regulating inflammation (26). Numerous experimental and epidemiological studies have implicated obesity-related adipose dysfunction as one of the main causes of endothelial dysfunction (27). Adipocyte dysfunction often leads to activation of NLRP3, and its activation leads to caspase-1 activation and the release of the inflammatory cytokines interleukin 1(IL-1) and interleukin 18 (IL-18) (28). These two inflammatory factors increase monocyte chemo-attractant protein-1 (MCP-1) and vascular cell adhesion molecule 1 (VCAM-1), thereby promoting leukocyte-endothelial cell adhesion, causing endothelial dysfunction. Endothelial cell dysfunction, as demonstrated by increased production of endothelial cell adhesion molecules and pro-inflammatory mediators, results in infiltration of monocytes in the subendothelial layer (29). Under the influence of macrophage colony-stimulating factor, these monocytes transform into macrophages and engulf non-biodegradable oxidized LDL cholesterol, ultimately transforming into foam cells, leading to the development of atheromatous plaques (30).

Exogenous H_2_S has been shown to inhibit high glucose-induced activation of the NLRP3 inflammasome in adipocytes. In the study by Hu et al., high glucose was found to induce the up-regulation of NLRP3 in adipocytes, which promoted the release of downstream molecules IL-1β and IL-18. The above phenomenon was found to be inhibited by exogenous sodium hydrosulfide (16). This suggests a potential anti-atherosclerotic effect of exogenous H_2_S in obese patients, especially those with adipose tissue dysfunction. However, further studies are required for in-depth characterization of the molecular mechanisms underlying the anti-inflammatory effect of H_2_S.

### H_2_S inhibits ICAM-1 expression on endothelial cells

ICAM-1 plays an important role in immune and inflammatory responses, including atherosclerosis. These proteins are ligands for the leukocyte adhesion protein LFA-1 (integrin alpha-L/beta-2). During leukocyte trans-endothelial migration, ICAM1 engagement promotes the assembly of endothelial apical cups through activation of ARHGEF26/SGEF and RHOG, eventually leading to trans-endothelial migration of leukocytes (31). ICAM-1 in the blood has been recognized as a marker of vascular inflammation in atherosclerosis; it has been shown to predict cardiovascular risk and future cardiovascular disease (32).

The study of H_2_S and ICAM-1 was originally reported in the context of non-steroidal drug therapy. H_2_S was found to reduce the increase in ICAM-1 caused by non-steroidal anti-inflammatory drugs (33). Subsequent studies found that exogenous H_2_S can slow down the expression of ICAM-1 in the blood of apoE knockout mice. In addition, under the influence of exogenous sodium hydrosulfide, the size of arterial plaque also decreased (20). This phenomenon was attributed to the prevention of the activation of the nfkb signaling pathway by H_2_S.

### H_2_S inhibits hemoglobin oxidation and prevents lipid peroxidation

In the atherosclerotic lesions, a pathological change called “infiltration of red blood cells” is usually found (34). Some of the damaged red blood cells are phagocytosed by macrophages and degraded in lysosomes. As a product of lysosomal digestion, iron ions are excreted by macrophages by exocytosis, inducing the oxidation of LDL to OxLDL. Subsequently, the macrophages phagocytose OxLDL (35). In addition, degraded red blood cells release hemoglobin (Hb), which can react with surrounding plaque lipids. This leads to the formation of different oxidatively modified hemoglobin species, such as metHb (Fe^3+^) and ferrylHb (Fe^4+^ =O^2−^) (36). Oxidized Hb species sensitize vascular endothelial cells to oxidant-mediated killing, suggesting that it is a potential causative factor in atherosclerosis.

Potor et al. found that H_2_S significantly reduces oxidation of Hb preventing the formation of ferrylHb derivatives. By inhibiting Hb-lipid interactions, sulfide lowers oxidized Hb-mediated induction of adhesion molecules in endothelium and disruption of endothelial integrity (37). Chemically, H_2_S is a reductant, and there is evidence that H_2_S can convert oxidized low-density lipoproteins to lipoalcohols (38). This heralds the potential of H_2_S in inhibiting hemoglobin oxidation and preventing lipid peroxidation.

### H_2_S inhibits intravascular thrombosis

Platelets play an important role in the pathogenesis of coronary thrombosis and atherogenesis. Abnormal activation of platelets contributes to atherothrombosis (39). Activation of platelets induces the release of chemokines, leading to the aggregation of leukocytes, followed by the progress of leukocyte and platelet adhesion, which is mainly mediated by P-selectin and ligand PSGL-1 (40). In addition, degranulation of platelets leads to a release of inflammatory cells molecules, including various chemokines, cytokines, lipids, and proteins (41–43). The recruitment of leukocytes to the thrombus is a complex process. Specifically, leukocytes act by binding to p-selectin on the surface of platelets (44), rolling on the endothelium, and finally adhering to activated platelets. As a next step, leukocytes undergo integrin-mediated changes in shape and cellular functions such as motility, migration, degranulation, or phagocytosis. Almost at the same time, leukocytes promote platelet aggregation and secretion (45), enhance the production of thrombin and tissue factor (46, 47), and play an important role in the stability of the thrombus.

Previous studies have confirmed that H_2_S could inhibit platelet activation and aggregation (48–50). In the study by Grambow et al., exogenous H_2_S treatment was found to reduce platelet and leukocyte aggregation, and this inhibition showed a significant correlation with the concentration of H_2_S. The team then used scanning electron microscopy to directly analyze the specific effects of H_2_S on platelet activity, and found that the activated platelets in the control group exhibited changes in shape with formation of pseudopodia, leading to the appearance of a thorn apple-like shape under microscope. Cells treated with GYY4137 did not exhibit the above morphological changes, but instead were characterized by “round platelet morphology” and “less pseudopodia formation” (51). This phenomenon may be related to the inhibitory effect of H_2_S on the extracellular action of platelet p-selectin. Currently, there is a lack of direct evidence that H_2_S inhibits platelet activation, such as the lack of necessary rescue experiments to demonstrate that H_2_S has a direct effect on platelet p-selectin. However, the available evidence suggests the antithrombotic potential of this gas molecule, providing a new avenue of antagonism toward atherosclerotic and thrombotic diseases.

### H_2_S inhibits oxidative stress

Oxidative stress is recognized as a distinct factor in the pathogenesis of cardiovascular diseases. It is manifested specifically by an imbalance in the production and removal of oxygen free radicals in cells, with the production of some reactive oxygen species (ROS) such as O2^−^, OH^−^, ONOO^−^, and H_2_O_2_ (44). ROS can induce upregulation of endothelial cell adhesion molecules, proliferation and migration of vascular smooth muscle cells (VSMCs), platelet activation, lipid oxidation, and activation of matrix metalloproteinase, all of which can contribute to the advancement of atherosclerotic disease (52). The following mechanisms of the protective effect of H_2_S against oxidative stress have been identified: (1) it acts as a reducing agent to directly induce ROS scavenging *in vivo* (53); (2) protects proteins from oxygen radical attack by modulating the expression and activity of classic antioxidants, such as glutathione (GSH) and thioredoxin (Trx) (45); (3) regulates mitochondrial metabolism to limit ROS formation (46); (4) reduces ROS production by interacting with cytochrome c and providing electrons to the mitochondrial ATP synthesis mechanism, which can substitute oxygen in the ATP production mechanism (47).

Warnholtz et al. (54) established a hypertension model generated by AngII and found that AngII substantially enhanced superoxide anion generation in the aorta, which was reduced by NaHS therapy. Later, Hsin-Ying et al. discovered that exogenous H_2_S may suppress the IL-6-induced oxidative stress in rat vascular smooth muscle cells and decrease their ROS content (55). Notably, iNOS can sustainably produce NO under the continuous stimulation of ROS. Excessive NO release is seen as a potential risk factor for cardiovascular disease. H_2_S has been shown to reduce ROS release by IL-6 stimulation, thereby inhibiting the sustained increase in NO release by iNOS activation, ultimately delaying the phenotypic transformation of endothelial smooth muscle cells (55). This suggests that the anti-oxidative stress effect of H_2_S may be mediated *via* inhibition of the ROS-iNOS-NO pathway.

### H_2_S inhibits the proliferation of vascular smooth muscle cells

VSMCs are located in the medial layer of arteries and play an important role in the regulation of the vascular system (56). Physiologically, VSMCs possesses both systolic and diastolic phenotypes, regulating both vasoconstriction and relaxation. Under the stimulation of pathological conditions such as vascular injury, angiotensin II (Ang II), platelet-derived growth factor, insulin-like growth factor 1, ROS, and endothelin-1, the phenotype of cells undergoes a deconversion from a contractile phenotype to a synthetic phenotype, which ultimately leads to cell proliferation and migration (57–59). Proliferation of VSMCs is associated with various vascular diseases such as atherosclerosis, restenosis, and hypertension. In particular, this phenotypic switch plays an important role in atherosclerosis and plaque stability, and inhibition of vascular smooth muscle phenotype switch may be beneficial in advanced atherosclerosis (60). The myocardin serum response factor regulatory module, for example, is a critical component of phenotypic regulation because it permits the combinatorial interactions of activating and repressing signals and cofactors that operate on the majority of VSMC contractile genes. Compared to myocardin^+/+^ littermates, myocardin^+/−^ mice on an ApoE^−/−^ background showed increased atherosclerosis with greater concentration of macrophage or macrophage-like cells (61).

Earlier studies confirmed that the body transmitter H_2_S plays a broad role in the cardiovascular system. For instance, H_2_S was shown to induce a dose-dependent suppression of the proliferation of VSMCs through the MAPK pathway (62). With further advancement of research, H_2_S was found to inhibit the proliferation of VSMCs by regulating chromatin remodeling and target gene expression. Brg1 is the central catalytic subunit of the SWI/SNF apparatus (an ATP-dependent chromatin remodeling complex). Li et al. demonstrated that Brg1 plays an important role in the inhibition of VSMC proliferation induced by H_2_S by overexpressing and knocking out the *Brg1* gene. The effect of H_2_S on Brg1 was confirmed by luciferase reporter assay and real-time quantitative PCR at the transcriptional level. Finally, they used chromatin immunoprecipitation experiments to confirm that H_2_S inhibited the recruitment of Brg1 to the Pcna, Ntf3, and Pdgfα promoters, thereby acting as anti-VSMC proliferation (63). The above studies suggest that H_2_S regulates the proliferation of smooth muscle cells through epigenetic modification. Because epigenetic changes may be temporary or reversible, this provides a new idea for the development of late-stage drug therapy.

### H_2_S attenuates foam cell formation

Accumulation of foam cells is a hallmark pathological change in the development of atherosclerosis. Macrophages are an important source of membrane cell formation. Macrophages phagocytose cholesterol or oxidized LDL, and then esterify the above substances to extrude them from the cell. This is a normal compensatory phenomenon; however, disruption of this compensatory mechanism leads to excessive accumulation of lipids in cells, eventually leading to the formation of foam cells (64). Foam cell accumulation is involved in the development of atherosclerosis, such as the release of matrix-degrading enzymes, leading to plaque rupture or vascular occlusion (65).

Since macrophage phagocytosis of oxidized LDL is critical for foam cell formation, H_2_S has been reported to inactivate macrophage phagocytosis of LDL. This phenomenon can be reflected in the uptake of lipids by macrophages. In a study, the uptake rate of oxidized LDL was found to have significantly decreased in cells pretreated with sodium hydrosulfide (15). Further research found that the endogenous H_2_S inhibitor PPG could reverse the passivation effect of H_2_S and restore the efficiency of lipid uptake by macrophages. ACAT-1 is a kind of mitochondrially localized enzyme that catalyzes the reversible formation of acetoacetyl-CoA from two molecules of acetyl-CoA. Defects in this gene are associated with 3-ketothiolase deficiency (66). Currently, ACAT-1 is believed to be a key enzyme promoting the intracellular cholesterol accumulation within macrophages. In a study, NaHS was shown to reduce macrophage ACAT-1 expression and inhibit foam cell formation (15). This demonstrated that ACAT-1 may be the target of H_2_S to inhibit the transformation of macrophages into foam cells.

### H_2_S regulates cellular functions by S-sulfhydrating proteins

Although several studies have demonstrated the protective effect of H_2_S against atherosclerosis, its precise molecular mechanism is not clear. S-sulfhydration has recently been recognized as a major mechanism of the physiological effects of H_2_S, and several recent studies have explored H_2_S signaling by S-sulfhydration. S-sulfhydration modifies the functions of a specific protein by causing post-translational modifications. In a study, H_2_S was shown to increase keap1 protein thiolation, boost Nrf2 nuclear translocation, and limit ${\text{O}}_{2}^{-}$ production in endothelial cells, and this effect was abolished when Keapl was mutated at Cys151, but not Cys273, in endothelial cells (67). The enzymatic process involved in the synthesis of H_2_S in mammalian tissues includes cystathionine γ-lyase (CSE) and cystathionine β-synthase (CBS) (68). A study found that H_2_S donor treatment can lead to thiolation of C252, C255, C307, and C310 of CSE, promote its binding to L-hcy, and prevent hyperhomocysteinemia-induced atherosclerosis in mice (69).

Protein sulfhydrylation triggered by H_2_S causes proteins to have various effects. These findings shed light on the specific molecular mechanism of the anti-antherosclerotic effect of H_2_S. Thus, H_2_S- induced protein sulfhydrylation is a potential promising topic for future research on this subject.

## Advantages and limitations of various anti-atherosclerotic H_2_S donor designs

Hydrolysis-triggered, intestinal flora metabolism-triggered, biothiol-triggered, esterase-triggered, pH-triggered, and other mechanisms are used by the current H_2_S donors with anti-AS properties. The pharmacokinetic features of the donors are determined by the various triggering mechanisms, which are critical for their continued development as prodrugs.

### Inorganic sulfides

Many inorganic sulfide salts have been utilized to synthesize H_2_S donors, including Na_2_S, NaHS, and CaS. Water usually produces these inorganic salts, such as Na_2_S_9_H_2_O, which are often used in laboratories. Dissolution of inorganic sulfate in water gradually leads to a state of chemical equilibrium between S_2_, HS, and H_2_S. The specific reaction formula is as follows: NaHS + H_2_O NaOH + H_2_S↑ (70). The benefit is that H_2_S is rapidly produced after hydrolysis and no by-products are formed during the chemical reaction, essentially eliminating bias in experimental findings caused by by-product activity. This is why it holds tremendous promise for research on use of exogenous H_2_S in the treatment of AS. Unfortunately, there are several major obstacles to developing inorganic sulfide salts as anti-AS prodrugs. First, it is difficult to determine the exact concentration of inorganic sulfate salts in solution due to the variety of purity levels used; secondly, inorganic sulfate salts are extremely volatile in aqueous solution following H_2_S hydrolysis, making it difficult to maintain the active ingredient during the configuration of the drug in clinical practice when intravenous drip treatment is used. Change of mode of administration to intravenous injection to reduce the impact of volatility of H_2_S may lead to an inordinately high concentration of H_2_S at the injection site, which is liable to lead to adverse effects. Finally, the produced H_2_S is rapidly diluted, making it difficult to sustain a reasonably extended period of time at the lesion. Due to the aforementioned deficiencies, the use of inorganic sulfide salt donors has been largely confined to animal experiments or cytohistological research.

### Allicin

Allicin is a naturally occurring compound in common foods such as onions and garlic. After digestion and absorption, allicin in certain foods can slowly produce H_2_S (71). Although allicin is a natural compound with a high safety profile and widespread availability, its decomposition products (DADS, DAS, and DATS) have low water solubility, slow rate of production of H_2_S, and a low pharmacokinetic profile, which frequently results in a lengthy test period. Second, interaction of allicin in the body leads to production of several by-products, making it impossible to identify the specific chemical responsible for anti-AS effects. These issues restrict the research on allicin as an H_2_S donor.

### GYY4137

Moore's team at the National University of Singapore published a report on Morpholinol thiosphosphonate, a water-soluble, long-acting H_2_S donor chemical, in 2008 (72). H_2_S produced by hydrolysis of this molecule is quite moderate when compared to inorganic sulfides. GYY4137 hydrolysis produces a peak H_2_S release of 10 min, while sodium hydrosulfide hydrolysis produces a peak H_2_S release of just 10 s. GYY4137 is progressively gaining favor among researchers because it seems to compensate for the unstable H_2_S emission during hydrolysis of inorganic sulfates. In a study by Qiu et al., diabetic model rats who were administered GYY4137 before myocardial ischemia-reperfusion injury showed smaller infarcts, reduced apoptosis, and lower oxidative stress than the control group, implying that the protective effect of GYY4137 against myocardial ischemia-reperfusion injury is linked to p-Akt and nuclear Nrf2 protein (73). In addition, Zheng et al. employed GYY4137 to show that H_2_S molecules regulate the PI3K/Akt/TLR4 signaling pathway to stabilize vascular plaques (74). As research continues, the atherosclerotic potential of GYY4137 is increasingly being unraveled, such as prevention of vascular inflammation and oxidative stress (75), protection against myocardial fibrosis (18), and attenuation of adverse remodeling (76).

Many researchers theorize GYY4137 as a beneficial research tool; however it does have some disadvantages. First, the end-product of GYY4137 is often sold as a dichloromethane complex, and one of the metabolites of dichloromethane is CO, another gaseous molecular signal with biological effects comparable to H_2_S. As a result, determining whether CO is involved in the biological effects of GYY3137 is challenging. Second, since the rate of hydrolysis of GYY4137 is exceptionally slow, greater dosages may be necessary to attain therapeutic H_2_S concentrations. This would increase the risk of adverse effects due to high doses of GYY4137. To the best of our knowledge, no extensive pharmacokinetic studies of GYY4147 have been conducted nor is there a reliable way to determine the quantity of H_2_S generated by hydrolysis of this compound (77). Because of the aforementioned flaws, GYY4137 has a limited role in the development of H_2_S prodrugs.

### NOSH-aspirin (NBS-1120)

NOSH-aspirin, is a synthetic derivative of 1,2-dithio-3-thione and aspirin with a nitrate moiety. It was developed as a potential substitute for the commonly used anti-platelet medication aspirin (78). Currently, NOSH-aspirin research is being largely conducted in the context of pancreatic cancer, colon cancer, neurodegeneration, antiplatelet, and anti-inflammatory, analgesic, and antipyretic (79). Despite the paucity of research on the development of this molecule as an anti-AS agent, its unique drug metabolism properties appear to hold a lot of promise. NOSH-aspirin can be broken down in the body into three different chemicals that have different physiological effects: H_2_S, nitric oxide, and aspirin (80). In clinical practice, aspirin is regarded the first-line anti-atherosclerotic drug, while the cardiovascular protective properties of nitric oxide (a gaseous molecular signal), have already been well-established (81, 82). In addition to the combination of H_2_S and nitric oxide, which functions as a stomach mucosal prostaglandin mimic and effectively suppresses the gastrointestinal adverse effects induced by aspirin, H_2_S may further boost the anti-atherosclerotic activity of aspirin (83). Although NOSH-aspirin is a promising H_2_S donor, its anti-atherosclerotic capabilities have not been well-investigated. Because all of the metabolites have anti-atherosclerotic properties, suitable compounds must be developed to assess the individual effects of each of the products, and further research is expected in the future.

### Thiol-triggered hydrogen sulfide donors

Thiol-triggered H_2_S donors are non-hydrolyzation-triggered H_2_S donors and were one of the first documented synthetic H_2_S donors. N-(Benzoylthio)benzamides are representative thiol-triggered H_2_S donors. The donor design is based on the instability of the S-N bond, which when broken releases H_2_S. The S-N bond is protected by an acyl group, which acts as a switch by regulating the chemical reaction of the acyl group, allowing the S-N bond to be exposed again and thus indirectly controlling the release of H_2_S (84). In addition, Acyl Perthiol Donors were also designed to be a mercaptan-triggered H_2_S donor. Acyl perthiol donors (RC(O)-S-SR) were first synthesized by Xian, where the R group is derived from penicillamine (85). The donor was synthesized from thiobenzoic acid derivatives and n-benzoyl cysteine methyl ester. Briefly, C- and N-protected penicillamine was first treated with 2,2 -dibenzothioazolyl disulfide to provide a penicillamine-benzothioazolyl disulfide intermediate, and then treated with corresponding thioacids to furnish the desired penicillamine-based donors. Subsequent experiments demonstrated that the donor could significantly reduce the area of myocardial ischemia-reperfusion injury, and the cytotoxicity test showed lower cytotoxicity of the donor compared to that of sodium hydride. Dithioperoxyanhydrides is another thiol-triggered donor of H_2_S. It is prepared in a single reaction step involving thiobenzoic acid and methoxycarbonylsulfenyl chloride (CH3OC(O)SCl) with fair overall yields. *In vivo* experiments confirmed that Dithioperoxyanhydrides can also induce total vasorelaxation of isolated rat aortic rings pre-contracted with phenylephrine (86). Other types of thiol-triggered H_2_S donors include Arylthioamides and S-aroylthiooximes. Studies have also demonstrated the potential for clinical application of these two kinds of donors. Arylthioamides were shown to strongly abolish the noradrenaline-induced vasoconstriction in isolated rat aortic rings and to hyperpolarize the membranes of human vascular smooth muscle cells in a concentration-dependent manner (87). In a study by Jeffrey et al., S-aroylthiooximes were found to significantly reduce the survival of HCT116 colon cancer cells relative to Na_2_S, GYY4137, and a small molecule SATO (88), indicating that this donor may inhibit smooth muscle proliferation and migration in atherosclerotic diseases.

### Enzyme-triggered H_2_S donors

The “trimethyl lock” lactonisation reaction, which entails cleavage of a phenolic ester by esterase, followed by spatial repulsion of the three methyl groups triggering lactonisation and the release of the drug from the adjacent carbonyl group, is the basis for the enzyme-triggered H_2_S donor design (89).

Enzymes are tissue- and substrate-specific active proteins that are found in all living organisms (90). Sofia-Iris Bibli demonstrated greater CSE expression in endothelial cells near carotid plaques than in endothelial cells in superior mesenteric arteries (91). We hypothesize that by combining the aforementioned qualities, enzyme-triggered H_2_S donors may be created to allow for the optimal concentration of H_2_S in the causative lesion, laying the groundwork for targeted drug therapy. Despite the paucity of research on enzyme-triggered H_2_S donors, enzyme-triggered design should be a trend in future pro-sulfide donor design.

### Specific PH-triggered H_2_S donors

Based on the poor targeting of many of the current mainstream H_2_S donors, Xian's team used intramolecular cyclisation to activate phosphorothioates to design a new pH-regulated release of H_2_S donor JK; JK series H_2_S donors have great potential for development in cardiovascular disease research, especially in the field of myocardial ischemia reperfusion injury. Due to myocardial ischemia or hypoperfusion, tissue hypoxia leads to accumulation of lactic acid, reducing the pH level in the responsible lesion and nearby tissues, which provides a basis for targeted therapy for this type of donor to release H_2_S (92). In addition, due to the donor's special mechanism of triggering the release of H_2_S, the protective effect of donors on organs in the context of gastric diseases is also well-reflected. In the study by Yang et al., JK donor significantly alleviated gastric mucosal damage, a side-effect of NSAIDs (93). Intragastric pre-administration of JK-1 was found to mitigate the side effects of NSAIDs, such as inflammatory cell infiltration, increased IL-6 and TNF-α release, and oxidative loss. Increase in CBS and CSE levels is a well-known requirement for *in vitro* osteogenic differentiation. JK has also been shown to promote osteogenic differentiation *in vitro* (94). In conclusion, JK has shown potential therapeutic value in other therapeutic fields besides cardiovascular diseases. These findings indicate that this compound is a H_2_S donor that is worthy of further research and development.

### Other types of donors

In addition to the types of H_2_S donors described above, there are other types of H_2_S donors, such as “UV-triggered H_2_S donors” and “carbonyl H_2_S donors” (95, 96). However, these donors have poor potential for the development of anti-AS pre-drugs. For example, UV-triggered H_2_S donors have weak penetration due to the short wavelength of UV light, making it impossible to trigger the donor drug deep in the tissue. Carbonyl H_2_S donors have poor water solubility and poor targeting. As a result, these donors are mostly used in *in vitro* assays.

## Summary and outlook

It has been more than 20 years since the H_2_S donor was successfully developed (97). Despite considerable breakthroughs in the research on H_2_S donors in the last two decades, targeting of the donor is still a key issue to be addressed. Clinical use of H_2_S as an anti-AS therapy depends on the design of a well-targeted donor and its delivery and release in the vicinity of the target lesion.

Nanomaterials were initially used to improve the targeting of antitumor drugs. The targeting of nanomaterials is mainly reflected in two aspects—active targeting and passive targeting. First, the alteration of ligands on the surface of nanoshells and the binding of ligands to receptors to provide precisely focused treatment is referred to as active targeting. Second, the diameter of the nanoparticles is more easily absorbed in some lesions due to passive targeting because of inadequate capillary endothelial connection in the microcirculation at the lesion site (e.g., in tumor tissue or inflammatory tissue). Due to the presence of inflammatory lesions near the arterial plaque lesions, the capillary endothelial junctions in the tissue are incomplete, and this forms the basis for passive targeting of nanomaterials. This implies that in normal tissues, drugs modified by nanomaterials are not easily absorbed but accumulate adjacent to the inflamed tissues, thereby achieving targeted therapy. Willem's team created unique multimodality HDL-mimicking nanoparticles by incorporating gold, iron oxide, or quantum dot nanocrystals for computed tomography, magnetic resonance, and fluorescence imaging, respectively (98), It is generally known that macrophage infiltration around arterial plaques is a specific pathophysiological change in AS. The team cleverly used this principle to design a plaque-targeted imaging drug. Moreover, a study by Wang et al. demonstrated good biocompatibility of macrophage membrane functionalized biomimetic nanoparticles: the biomimetic particles were rapamycin-loaded poly(lactic-co-glycolic acid) copolymers made from macrophage membrane coating. Such nanoparticles can be efficiently targeted and accumulated in atherosclerotic lesions *in vivo*. After a 4-week treatment plan, MM/RAPNPs were found to have significantly delayed AS progression (99).

Similar principles can be used to design vascular-targeted H_2_S donors based on the above research. Theoretically, this design idea offers three advantages: First, because surface proteins on the macrophage membrane provide good active targeting of H_2_S donors, especially α4β1, this receptor can tightly bind to vascular cell adhesion molecule-1 (VCAM-1) on the surface of endothelial cells, which is highly expressed in the inflamed endothelium. Second, nanotechnology-modified H_2_S donors are also passively targeted and can accumulate around inflammatory tissues near vascular plaques to achieve ideal therapeutic concentrations. The last point is also the most critical; since nanomaterials are foreign bodies, they often induce the activation of innate immunity. The above situation can be significantly avoided by using macrophage membranes as biomimetic coatings of nanomaterials (100, 101). This technology is poised to gradually mature in the near future.

In addition to drug targeting, several other important questions remain unanswered in the field of H_2_S donors, which require multidisciplinary collaboration involving chemistry, pharmacology, and biomaterials science. Firstly, there is considerable variability in the therapeutic concentration window for H_2_S molecules used to correct different pathophysiological states and even different stages of the same pathophysiological state. Therefore, clarifying the therapeutic window of H_2_S for specific targets is a crucial imperative. Secondly, as H_2_S is highly volatile and rapidly metabolized, the relationship between the effective dose of the drug and the donor dose is not necessarily a simple linear relationship. Therefore, it is crucial to define the release rate of the H_2_S for the development of H_2_S donors. In addition, whether the by-products or the donor itself can produce biological effects after the triggering of the H_2_S donor and bias the experimental results is also an important issue to be addressed. As the field of H_2_S research continues to evolve, these critical issues must be addressed in order to progress toward clinical treatment. Future multidisciplinary collaborations involving the disciplines of nanotechnology, chemical synthesis, drug metabolism, and biology may eventually make clinical pathways for H_2_S treatment possible.

## Author contributions

Z-SJ, Y-WY, and N-HD: manuscript conceptualization. Y-WY and N-HD: writing original manuscript draft. K-JT, L-SL, and ZW: literature search and articles acquisition. D-HW and H-TL: figures drawing. All authors contributed to the article and approved the submitted version.

## Funding

This work was supported by the National Natural Science Foundation of China (91839103 to Z-SJ), and Postgraduate Scientific Research Innovation Project of Hunan Province (CX20210958 to N-HD).

## Conflict of interest

The authors declare that the research was conducted in the absence of any commercial or financial relationships that could be construed as a potential conflict of interest.

## Publisher's note

All claims expressed in this article are solely those of the authors and do not necessarily represent those of their affiliated organizations, or those of the publisher, the editors and the reviewers. Any product that may be evaluated in this article, or claim that may be made by its manufacturer, is not guaranteed or endorsed by the publisher.
